# Creative behavior, psychopathology, and salience processing: a case–control study of Italian artists from the Florence Academy of Fine Arts

**DOI:** 10.3389/fpsyg.2025.1541458

**Published:** 2025-05-12

**Authors:** Giuseppe Pierpaolo Merola, Andrea Patti, Bernardo Bozza, Davide Benedetti, Giulia Minotti, Andrea Saverio Spagnuolo, Giulia Pitt, Vincenzo Pecoraro, Andrea Lenti, Gaia D’Anna, Niccolò Porcinai, Silvia Tafuni, Isotta Fascina, Andrea Ballerini, Valdo Ricca

**Affiliations:** ^1^Psychiatry Unit, Department of Health Sciences, University of Florence, Florence, Italy; ^2^Eating Disorder Clinic "Residenza Gruber", Bologna, Italy; ^3^Santagostino Medical Center, Bologna, Italy

**Keywords:** aberrant salience, psychosis, psychopathology, creativity, art

## Abstract

**Introduction:**

Creative behavior has been associated with psychopathological traits, particularly in the psychotic spectrum. Aberrant salience, a transdiagnostic feature of psychosis vulnerability, may influence the creative process. This study aimed to investigate differences between artists and non-artists in aberrant salience, creativity, personality traits, and psychopathology.

**Methods:**

The sample consisted of 123 adults (58 artists, 65 controls) who completed self-report measures, including the Aberrant Salience Inventory (ASI), Big Five Inventory (BFI), Obsessive Beliefs Questionnaire (OBS), Remote Associates Test (RAT), and Anagram Task (ANAG). Statistical analyses included Mann–Whitney U tests for group comparisons, Spearman correlations, and regression analyses.

**Results:**

Artists showed significantly higher aberrant salience, openness to experience, and obsessive beliefs, with lower scores on the RAT and ANAG compared to controls. Regression analyses revealed that higher ASI scores were significantly predicted by greater Openness to experience, lower Conscientiousness and higher religiosity.

**Discussion:**

These findings suggest that artists have a greater propensity for altered salience experiences, which may contribute to their creative endeavors. The strong association between aberrant salience and openness to experience indicates that personality traits significantly influence creative expression and psychosis vulnerability. Religiosity’s role in predicting aberrant salience highlights the impact of cultural and spiritual beliefs on perceptual experiences. By identifying these associations, this study contributes to evaluating risk populations for psychosis. Artists exhibiting high aberrant salience may represent a subgroup with heightened vulnerability, underscoring the importance of early detection and intervention strategies within creative communities.

## Introduction

Creativity is a popular concept interpreted by various disciplines, but it still lacks a well-defined, unique definition. In general, creative behavior is described as the ability to generate ideas or solutions that are both novel and valuable (Green et al., 2024). The product created can be either a physical object or something more intangible, such as a theory or a form of expression. Expressive behavior results from the interplay of various domains, including deduction, intuition, reason, imagination, emotion, reflection, and divergent/convergent thinking (Javaid and Pandarakalam, 2021).

Though desirable, creativity has been linked to psychopathological traits since ancient times. Plato described it as “divine madness,” and Aristotle noted that many creative individuals exhibited “melancholia” (Becker, 2001). Empirical evidence of this link emerged in the second half of the 20th century (Heston, 1966; Karlsson, 1970), prompting new studies on the incidence of psychopathology in highly creative individuals (Knudsen et al., 2019; Post, 1994; Preti and Vellante, 2007). The literature thus showed an increased risk for schizophrenia and bipolar disorder (Kyaga et al., 2011; Kyaga et al., 2013).

Subsequent studies have investigated connections between creativity and major brain networks (Beaty et al., 2019). Among these networks, the salience network (SN) represents an altered function in psychosis spectrum disorders. Salience can be defined as the process by which stimuli or internal thoughts are attributed heightened significance or importance (Kapur, 2003). The most accredited model for psychosis involves the abnormality of this process, suggesting the presence of “Aberrant Salience” (AS), defined as the assignment of increased meaning to otherwise insignificant facts and details. AS appears to be a transdiagnostic trait-like feature of general vulnerability to psychosis, which usually remains stable over time (Lelli et al., 2013; Poletti and Sambataro, 2013). Biologically, it has been described how dysregulated dopaminergic transmission leads to stimulus-independent dopamine release; this neurochemical aberration alters context-driven attribution of salience and leads to misalignments of external objects and internal representations (Kapur, 2003). This process may share cognitive mechanisms with reduced latent inhibition, a construct that has been linked to both creativity and psychopathology (Carson, 2014). A person would have to pay attention to irrelevant details before assigning them salience, reinforcing the potential connection between aberrant salience and cognitive disinhibition.

While creative cognition and psychopathologies share traits like cognitive disinhibition and altered states of consciousness, the overlap is incomplete. It is possible that certain creative attitudes or approaches act as protective factors, mitigating or even reversing the risk of psychopathology (Carson, 2011).

Building on our previous findings (Patti et al., 2024), we observed that artists exhibited significantly higher aberrant salience scores compared to a control group. These results provided preliminary evidence that aberrant salience may contribute to creative cognition, a notion that informs the current investigation.

Thus, this study aims to explore potential correlations between the creative process and vulnerabilities associated with the psychotic spectrum. The primary focus is to investigate risk factors for aberrant salience (and thus psychosis) in populations identified as being at elevated risk, namely artists (MacCabe et al., 2018). Additionally, the study seeks to examine the relationship between psychopathological traits and creative behavior. By integrating these elements, the research aims to illuminate the mechanisms through which creativity and psychopathology intersect.

## Methods

### Participants

In this study, two groups of Italian adults were recruited: a control group and a group of artists from the Florence Academy of Fine Arts. The control group was recruited from the local university using convenience and snowball sampling methods, while the artists were specifically recruited from the academy. The inclusion criteria for both groups were age between 18 and 40 years, being Italian speakers, and providing written informed consent. The exclusion criteria for both groups included illiteracy and the inability to provide consent or complete the survey. The same questionnaire and creativity tests were administered to both groups in person, under controlled laboratory conditions, ensuring consistent procedures and anonymity standards. The study was conducted over a period of seven months, from May 2023 to May 2024. The anonymity of participants was always ensured, and written informed consent was collected before enrollment. The study procedure was approved by the local University Ethical Committee (code 893197).

### Measures

Self-reported data encompassed socio-demographic details, personal or familial self-report of psychiatric conditions. In the artists group, the main artistic interests were recorded (up to three per participant).

The primary measure was the Aberrant Salience Inventory (ASI), a validated self-report scale assessing the propensity for aberrant salience attribution, a hallmark of psychosis spectrum disorders (Cicero et al., 2010). The ASI evaluates behavioral manifestations of altered salience processing, which are relevant to understanding the impact of psychosis-related conditions on behavior. It is a 29-item self-report scale with binary responses (“Yes”/"No”), evaluated alterations in salience. The ASI has demonstrated robust internal consistency, with Cronbach’s alpha values of 0.91 in psychotic populations and 0.80 in controls. The ASI scores vary from 0 to 29, where higher scores indicate increased psychosis vulnerability; the best cutoff for psychosis has been identified to be 14 (Merola et al., 2023). Recent meta-analytical findings have redefined its structure into three subscales: sensory sharpening, unveiling experiences, and heightened interpretation and emotionality (Merola et al., 2023).

The Big Five Inventory (BFI, Ubbiali et al., 2013) is a tool used to assess the five major dimensions of personality, based on the Big Five personality model. It includes 44 items. The BFI demonstrates satisfactory internal consistency, with Cronbach’s alpha values ranging from 0.69 to 0.83 across subscales. This inventory is designed for quick self-reporting while still effectively measuring the broad spectrum of personality traits.

The Italian version of the Obsessive Beliefs Questionnaire (OBS, Obsessive Compulsive Cognitions Working Group, 2003) evaluates central cognitive domains associated with obsessive-compulsive symptoms using a 46-item scale with a 7-point Likert response format. The subscales of the OBS include Perfectionism, Responsibility for Damage, Control of Thoughts, Responsibility for Omission, and Importance of Thoughts. Internal consistency for the subscales in control cases ranges from 0.82 to 0.93, indicating good to excellent reliability across all dimensions.

The Remote Associates Test (RAT, Salvi et al., 2020) is a creativity test used to measure the ability to form associations between seemingly unrelated concepts. Participants are given three words (e.g., “cream,” “skate,” “water”) and must find a fourth word (“ice”) that connects them all. This test evaluates the participant’s creative and associative thinking abilities. The test is scored based on the number of correct associations made within a time limit. The RAT, available in multiple languages, has demonstrated excellent internal consistency, with a Cronbach’s alpha of 0.89.

In the Anagrams Test (ANAG, Salvi et al., 2020) participants are presented with a single word at a time and must rearrange its letters to form a different word (anagram). This test evaluates cognitive flexibility, lexical retrieval, and verbal fluency, providing insights into linguistic processing and creative problem-solving abilities. The test scores are based on the number of the words formed. The ANAG also shows excellent internal consistency, with a Cronbach’s alpha of 0.92.

### Power analysis

An *a priori* power analysis was conducted to determine the sample size required for comparing the distributions of variables between artists and controls using the Mann–Whitney U test (McKnight and Najab, 2010). The analysis assumed two-tailed tests, a significance level (alpha) of 0.05, a desired power of 0.80, and an effect size of 0.6, which corresponds to a medium effect size according to Cohen’s benchmarks (Muller, 1989).The power analysis indicated that a sample size of 44 participants per group was required to achieve the desired power. Therefore, the total sample size needed for the study was 89 participants.

### Statistical analyses

To compare the distributions of the variables between the artists and control groups, a Mann–Whitney U test was performed. This non-parametric test was chosen to account for potential violations of normality assumptions and the ordinal nature of some variables. The characteristics of the sample were summarized as descriptive statistics (mean and SD reported, Table 1).

**Table 1:** Descriptive statistics.

	Mean (SD), artists	Mean (SD), controls	T-statistic	*P*-value (FDR corrected)
Gender **	0.266 (0.445)	0.534 (0.503)	1,357	0.0045
Age ***	22.594 (4.038)	29.966 (7.7)	327	< 0.001
Years of education ***	15.0 (2.817)	20.07 (1.888)	237	< 0.001
Religion	0.36 (0.485)	0.328 (0.473)	1,497	0.8314
Psychiatric condition (self reported)	0.231 (0.425)	0.121 (0.329)	2092.5	0.166
Psychiatric condition in family (self reported)	0.369 (0.486)	0.362 (0.485)	1898.5	0.9798
Psychotropic drugs (self reported)	0.077 (0.269)	0.103 (0.307)	1835	0.7531
RAT ***	15.93 (7.377)	22.714 (5.017)	359.5	0.0002
ANAG **	13.143 (4.773)	16.5 (4.481)	557	0.0027
ASI ***	20.446 (4.828)	10.0 (6.0)	3,397	< 0.001
OBS **	151.538 (44.842)	122.069 (43.977)	2,582	0.0013
Neuroticism	3.281 (0.793)	2.945 (0.857)	2326.5	0.0505
Extraversion	3.367 (0.823)	3.373 (0.734)	1879.5	0.9798
Agreeableness	3.655 (0.617)	3.728 (0.577)	1720	0.5379
Conscientiousness ***	3.2 (0.744)	3.696 (0.712)	1,157	0.0008
Openness ***	4.282 (0.491)	3.627 (0.719)	2,874	< 0.001

Mann Whitney U test between artists and controls (FDR corrected). Asterisks underline a statistically significant difference (*p* < 0.05: *; *p* < 0.01: **; *p* < 0.001: ***).

To explore the relationships between the variables, a Spearman correlation analysis was conducted. The analysis was performed separately for the artists group and the control group. The correlation analysis was corrected for age, gender and education, and the it involved the following variables of interest: ASI, OBS, Neuroticism, Extraversion, Agreeableness, Conscientiousness, Openness, Group (binary variable indicating whether the participant belonged to the artists group or the control group), RAT, ANAG. *p*-values were corrected for FDR.

The regression analysis aimed to identify key predictors of ASI scores in the artists group. Two models were tested: the first examined demographic variables (age, gender, education, religiosity, and family psychiatric history), and the second focused on the five personality dimensions from the Big Five Inventory (Neuroticism, Extraversion, Agreeableness, Conscientiousness, and Openness).

A stepwise regression approach was then used to select the best combination of five predictors from the total aforementioned 10 variables. The final model was evaluated for assumptions such as linearity, multicollinearity, and normality of residuals, with fit assessed using R^2^ values. The computations were conducted in Python 3.1.

## Results

### Comparison of the two groups

A total of 123 subjects were recruited, 65 within the control group and 58 in the artists group. Overall, 53% of the sample identified as female (57, 33% among controls and artists respectively), while the remaining portion identified as male. No participants in any group identified as non-binary, transgender, or chose to self-describe their gender. The average age was 27.42 ± 6.511 (controls 29.97 ± 7.7; artists 22.59 ± 4.038). Years in school averaged 15.40 ± 3.662 (controls 20.07 ± 1.888; artists 15.0 ± 2.817). In the artists group, participants’ main interests were the following: sculpture (68%), painting (54%), music (31%), illustration (18%), graphic design (12%), and others (5%).

Comparison of the two groups revealed that artists exhibited significantly higher ASI scores compared to controls (artists: 18.5 ± 4.3; controls: 10.2 ± 3.7; *p* < 0.001), suggesting an elevated vulnerability to psychosis-related aberrant salience. The Mann–Whitney test results are displayed in Table 1.

### Correlations between variables

**Figure 1:**
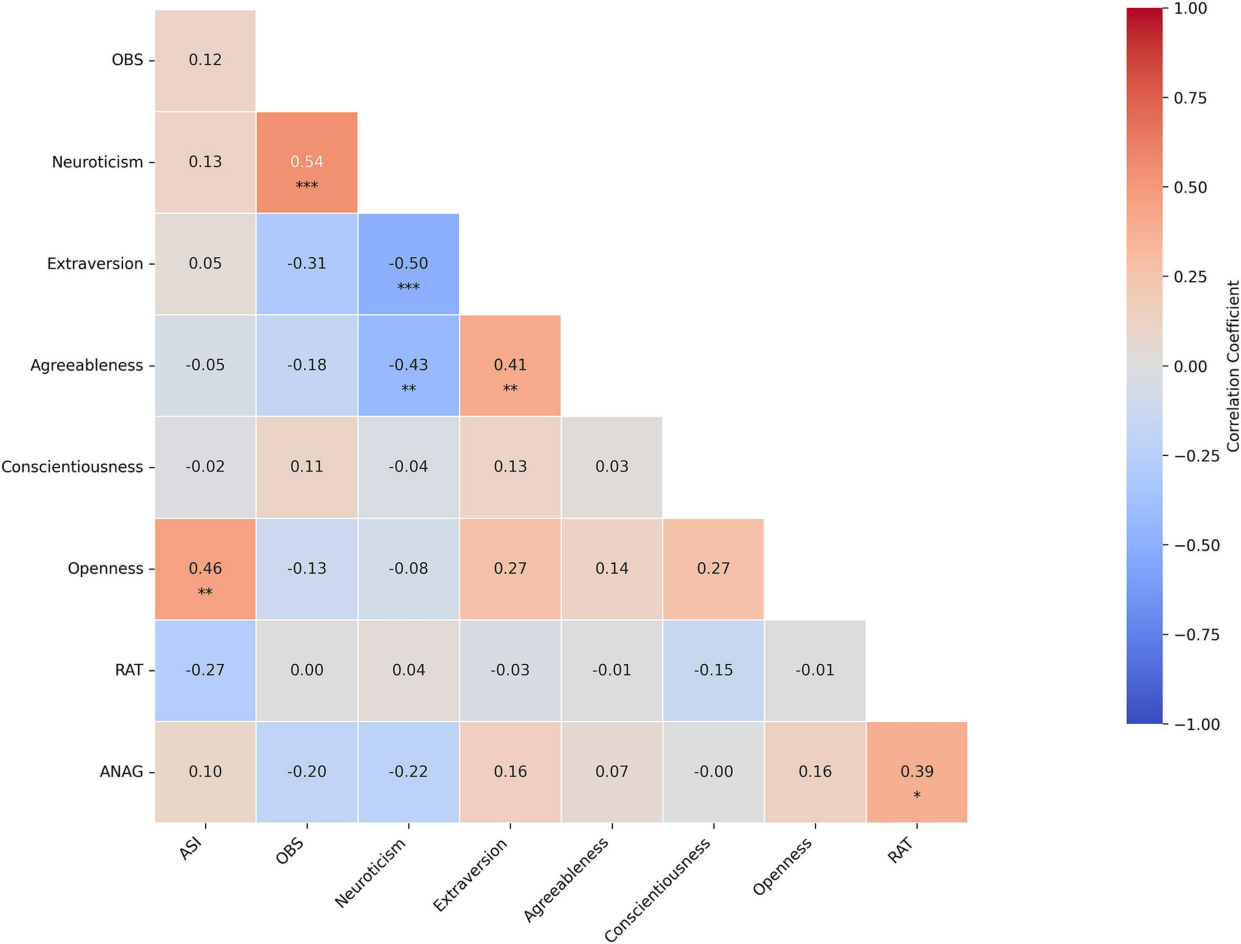
Partial correlations among psychological traits and creativity measures in the artists group, adjusted for age, gender, and education. Each cell displays the correlation coefficient. Asterisks underline a statistically significant difference (*p* < 0.05: *; *p* < 0.01: **; *p* < 0.001: ***), under FDR adjustment.

**Figure 2:**
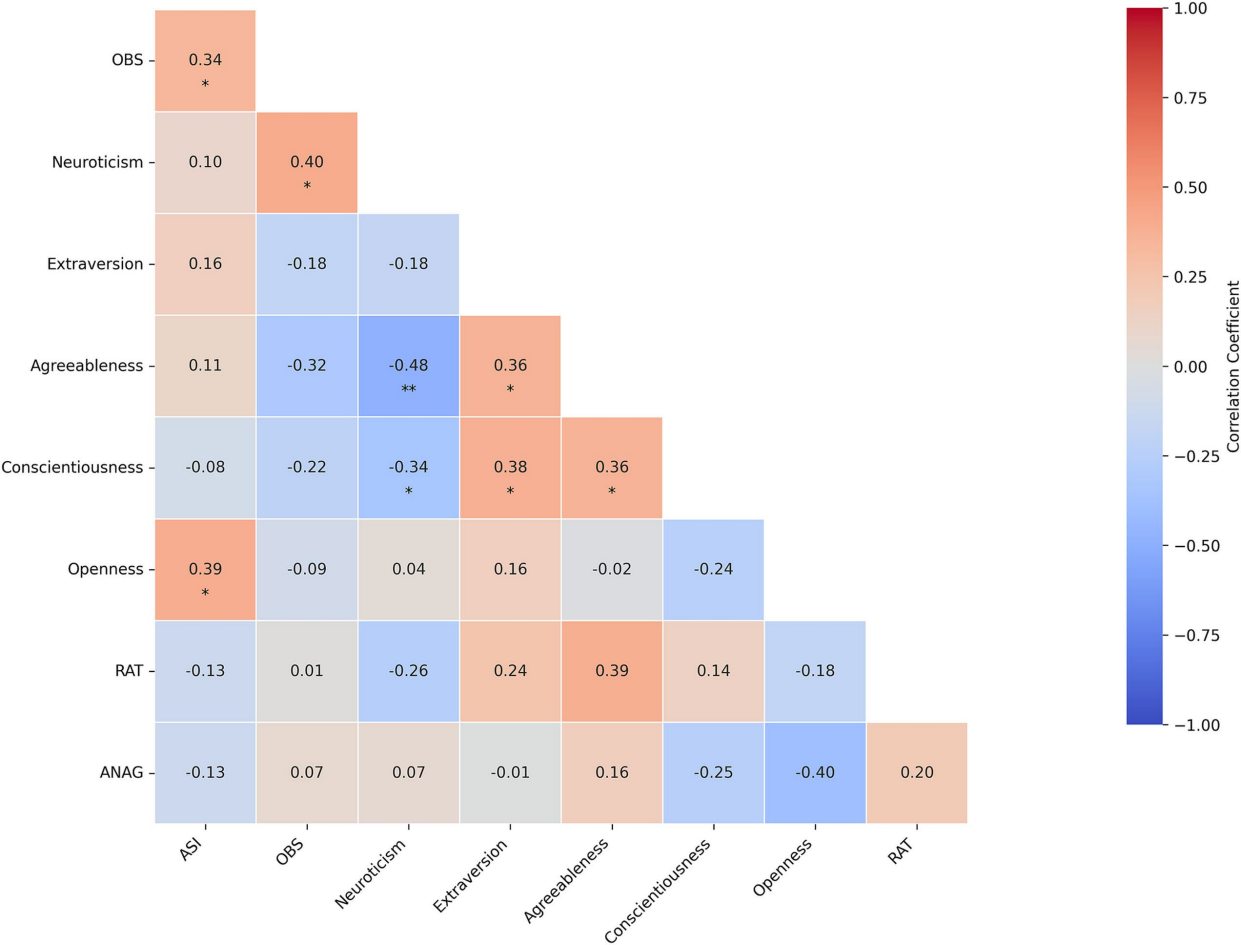
Partial correlations among psychological traits and creativity measures in the controls group, adjusted for age, gender, and education. Each cell displays the correlation coefficient. Asterisks underline a statistically significant difference (*p* < 0.05: *; *p* < 0.01: **; *p* < 0.001: ***), under FDR adjustment.

Partial correlation analysis with gender, age, and education as covariates (and FDR correction) revealed several significant results (see Figures 1, 2). In the artist group, ASI was significantly positively correlated with Openness (*r* = 0.46, *p* = 0.0012). OBS showed a significant positive correlation with Neuroticism (*r* = 0.54, *p* < 0.001). Neuroticism was significantly negatively correlated with Extraversion (*r* = −0.50, *p* < 0.001) and Agreeableness (*r* = −0.43, *p* = 0.00281). Extraversion and Agreeableness exhibited a significant positive correlation (*r* = 0.41, *p* = 0.00429). Additionally, RAT was significantly positively correlated with ANAG (*r* = 0.39, *p* = 0.0231). See Supplementary Table 1 for more details.

In the control group, ASI showed significant positive correlations with OBS (*r* = 0.34, *p* = 0.0358) and Openness (*r* = 0.39, *p* = 0.0177). OBS was significantly positively correlated with Neuroticism (*r* = 0.40, *p* = 0.015). Neuroticism exhibited a significant negative correlation with Agreeableness (*r* = −0.48, *p* < 0.001) and Conscientiousness (*r* = −0.34, *p* = 0.0358). Extraversion was significantly positively correlated with Agreeableness (*r* = 0.36, *p* = 0.0283) and Conscientiousness (*r* = 0.38, *p* = 0.0247). Agreeableness also showed a significant positive correlation with Conscientiousness (*r* = 0.36, *p* = 0.0283). More details can be found in Supplementary Table 2.

A partial correlation analysis was conducted on the entire sample as well, controlling for age, gender, and education (see Supplementary Figure 1). Significant associations were found between ASI and both Openness and OBS.

### Regression models

The regression models’ results are shown in Table 2. The first model, which focused on age, gender, education, religiosity, and family psychiatric history, explained 24.5% of the variance in ASI (*R*^2^ = 0.245, adjusted *R*^2^ = 0.181; *F* = 3.831, *p* = 0.005). Significant predictors included religion (*β* = 3.755, *p* = 0.005), family psychiatric history (*β* = 2.816, *p* = 0.022), years of education (*β* = −0.625, *p* = 0.018), and age (*β* = 0.356, *p* = 0.022). Gender showed a marginal effect (*β* = −2.210, *p* = 0.089).

**Table 2:** Regression analysis of demographic and personality variables on ASI and best-fit model.

Demographic variables R-squared: 0.245, Adjusted R-squared: 0.181, F-statistic: 3.831, *p*-value: 0.005			
	Coefficient	Standard error	*p*-value
Religion	3.755	1.299	0.005
Psychiatric condition in family (self reported)	2.816	1.194	0.022
Years of education	−0.625	0.257	0.018
Gender	−2.210	1.280	0.089
Age	0.356	0.151	0.022

Personality variables R-squared: 0.282, Adjusted R-squared: 0.221, F-statistic: 4.639, *p*-value: 0.001			
	Coefficient	Standard error	*p*-value
Neuroticism	0.944	0.786	0.234
Openness	5.211	1.174	0.000
Agreeableness	−0.245	0.988	0.805
Extraversion	0.053	0.776	0.946
Conscientiousness	−1.490	0.748	0.051

Best-fit after stepwise regression R-squared: 0.340, Adjusted R-squared: 0.284, F-statistic: 6.086, *p*-value: 0.000			
	Coefficient	Standard error	*p*-value
Religion	2.388	1.200	0.051
Psychiatric condition in family (self reported)	1.785	1.091	0.107
Conscientiousness	−1.511	0.716	0.039
Neuroticism	0.974	0.645	0.136
Openness	4.729	1.111	0.000

The second model, based on the Big Five personality traits, explained 28.2% of the variance (*R*^2^ = 0.282, adjusted *R*^2^ = 0.221; *F* = 4.639, *p* = 0.001). Significant predictors included Openness (*β* = 5.211, *p* < 0.001), with Conscientiousness almost reaching significance (*β* = −1.490, *p* = 0.051). Other traits, including neuroticism, agreeableness, and extraversion, were non-significant.

The stepwise regression combining variables from both models improved the fit (*R*^2^ = 0.340, adjusted *R*^2^ = 0.284; *F* = 6.086, *p* < 0.001). Key predictors included Openness (*β* = 4.729, *p* < 0.001) and Conscientiousness (*β* = −1.511, *p* = 0.039), with religiosity showing a trend (*β* = 2.388, *p* = 0.051).

## Discussion

The significant elevation of ASI scores among artists suggests that this group may have a heightened vulnerability to psychosis-related aberrant salience, which has important implications for behavior. The elevated AS in artists may reflect a unique interaction between creative processes and underlying neurobiological mechanisms associated with psychosis. By exploring this relationship, the study offers insights into the behavioral manifestations of psychosis-related conditions.

While controls show AS levels below the psychosis risk cut-off (score of 14), the artists demonstrated much higher levels. Artists did not report a higher prevalence of psychiatric history than controls (see Table 1). A plausible explanation is that creative individuals naturally exhibit a stronger tendency for altered salience, which may be integral to the creative process, particularly during periods of active creation (Patti et al., 2024). Moreover, being part of a supportive community, such as the Academy of Fine Arts, may alleviate self-stigma and reduce the risk of alienation (Mosher et al., 1995). ASI showed a significant positive correlation with Openness in both groups (the effect is more prominent among artists), suggesting that heightened openness to novel experiences is linked to the attribution of aberrant significance to stimuli.

The higher levels of Openness to experiences within the artists are in line with previous studies (Chamorro-Premuzic et al., 2009; Furnham et al., 2013; McCrae and Greenberg, 2014). Openness to experiences involves a broad appreciation for creative behavior, emotion, exploration, unconventional ideas, curiosity, and diversity of encounters. Individuals high in Openness tend to be receptive to emotions, more inclined to entertain unconventional viewpoints, appreciative of beauty, and willing to experiment with novel concepts.

Similarly, it is unsurprising that artists exhibit lower levels of Conscientiousness, which reflects a preference for spontaneous and flexible actions, such as artistic endeavors, over structured and planned behavior (Feist, 1998).

While Neuroticism is higher among artists, the difference does not reach statistical significance. This trait, also referred to as emotional instability, denotes a tendency to experience intense negative emotions, such as anger, anxiety, or depression, alongside heightened reactivity to emotional stimuli and vulnerability to stressors.

Artists showed higher levels of Obsessive Beliefs compared to controls. Available literature on the matter provides ambiguous results: while older studies argued that creativity does not vary as a function of obsessionality (Binik et al., 1981), more recent research suggested that obsessive symptomatology may contribute to creative behaviors (Wendler and Schubert, 2019).

Another notable finding is that artists scored lower on the RAT, a widely used measure of creativity. The RAT is generally considered a test of convergent thinking, which underpins traditional insight-based problem-solving (Huang, 2017; Lee et al., 2014; Lee and Therriault, 2013). Rather than interpreting the lower RAT scores among artists as evidence of diminished creative behavior, we propose they signify a reduced reliance on convergent thinking processes. This hypothesis is further supported by their similarly lower performance on the Anagram Task, a complementary measure of creativity that specifically assesses convergent problem-solving abilities (McBride et al., 2021).

Interestingly, some studies indicate that solving RAT problems also involves divergent thinking, adding complexity to these findings (Wu et al., 2017). These results suggest that the higher Openness to Experience and heightened sensory-perceptual receptivity, as indicated by elevated ASI scores in artists, may not align with convergent thinking-based creativity but instead point to a stronger reliance on divergent thinking processes. However, the lack of validated measures of divergent thinking in Italian at the time of this study limits definitive conclusions, warranting further research to explore this relationship.

The regression analyses reveal that both personality traits and demographic factors significantly predict aberrant salience. High Openness to Experience is associated with increased ASI scores, suggesting that individuals who are more open and receptive to new experiences may be more prone to perceiving unusual or significant patterns in their environment. Lower Conscientiousness is also linked to higher ASI scores among artists, indicating that less organized or disciplined individuals might be more susceptible to these perceptions.

Religiosity and a family history of psychiatric conditions emerge as significant predictors as well. Individuals with strong religious beliefs might be more inclined to assign profound meaning to everyday events, interpreting them through a spiritual or supernatural lens. This tendency can enhance the perception of aberrant salience. However, some ASI items assess religious, mystical, or spiritual experiences, which may introduce some confounding and make this hypothesis difficult to test.

Several limitations of the current study need to be acknowledged. Firstly, relying on self-reported measures may introduce the risk of either underreporting or overreporting subjective experiences. Second, our sample predominantly consists of individuals of European descent from Italy, which may limit the cross-cultural generalizability of our findings. Third, while we accounted for covariates in our analyses, the absence of a well-matched control group remains a methodological limitation that future research should address to enhance the robustness of comparisons. Lastly, the lack of a validated Italian divergent thinking test constrained our creativity measures, which we addressed by selecting RAT and ANAG; future studies could enhance cognitive assessments by incorporating validated divergent thinking tasks once available in Italian or other relevant contexts.

This study underscores the elevated levels of aberrant salience among artists, indicating a greater vulnerability to psychosis spectrum disorders and their behavioral manifestations. By linking heightened AS to personality traits and demographic factors, the research advances our understanding of the underlying mechanisms of psychosis and its impact on behavior. These findings highlight potential pathways through which pathological conditions influence behavior, offering avenues for future research and intervention strategies aimed at mitigating the behavioral impact of psychosis.

### Scope statement

The study investigates the intersection of creative behavior, psychopathological traits, and aberrant salience—a transdiagnostic marker of psychosis vulnerability—providing insights into the behavioral and neuropsychological underpinnings of psychosis-related conditions.

The investigation examines key constructs such as aberrant salience, personality traits, and creativity using validated psychometric tools, emphasizing their relevance in the psychotic spectrum and behavioral neuroscience. By focusing on artists, a group with a heightened propensity for altered salience experiences, we extend the understanding of how these traits manifest in creative communities, which are both highly relevant to psychosis research and behavioral conditions. Our findings contribute to identifying risk factors for psychosis, highlighting the roles of personality and cultural influences.

The manuscript offers valuable implications for early detection and intervention strategies in populations at risk for psychosis, aligning with the journal’s emphasis on translational neuroscience. Additionally, our research enhances the understanding of creativity and its neurobiological links to psychopathology, contributing to the broader discourse on behavioral conditions within the journal’s scope.

## Data Availability

The database of the studies, with the extracted data items, can be shared upon reasonable request to the corresponding author.
