# Supplementary figures and images for: Creative behavior, psychopathology, and salience processing: a case–control study of Italian artists from the Florence Academy of Fine Arts
Source: Front Psychol. 2025 May 12;16:1541458. doi: 10.3389/fpsyg.2025.1541458 (PMC12104814; doi:10.3389/fpsyg.2025.1541458)

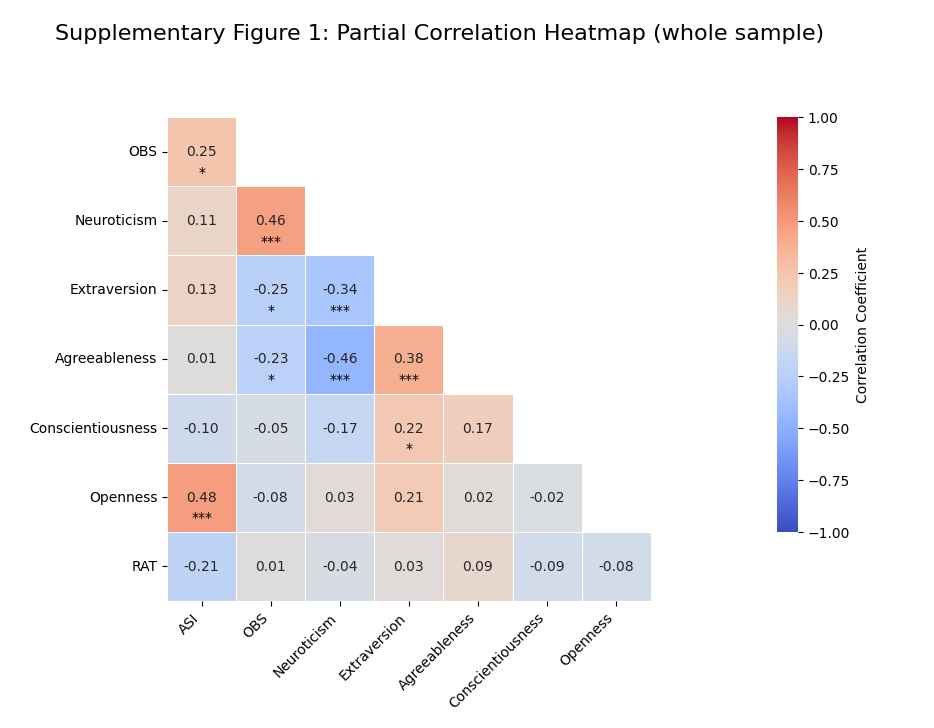

Supplement: Supplementary file 3 [file Image_1.png]
